# Measures of women's empowerment based on individual-level data: a literature review with a focus on the methodological approaches

**DOI:** 10.3389/fsoc.2023.1231790

**Published:** 2023-09-14

**Authors:** Janaína Calu Costa, Ghada E. Saad, Franciele Hellwig, Maria Fatima S. Maia, Aluísio J. D. Barros

**Affiliations:** ^1^International Center for Equity in Health, Universidade Federal de Pelotas, Pelotas, Brazil; ^2^Faculty of Health Sciences, American University of Beirut, Beirut, Lebanon

**Keywords:** women's empowerment, gender role, review, methods, measurement methods, girls and women, gender equality

## Abstract

**Background:**

Quantifying women's empowerment has become the focus of attention of many international organizations and scholars. We aimed to describe quantitative indicators of women's empowerment that are based on individual-level data.

**Methods:**

In this scoping review, we searched PubMed, Scopus, Web of Science, Science Direct, Google, and Google Scholar for publications describing the operationalization of measures of women's empowerment.

**Results:**

We identified 36 studies published since 2004, half of them since 2019, and most from low- and middle-income countries. Twelve studies were based on data from the Demographic and Health Surveys and used 56 different variables from the questionnaires (ranging from one to 25 per study) to measure the overall empowerment of women 15–49 years. One study focused on rural women, two included married and unmarried women, and one analyzed the couple's responses. Factor analysis and principal component analysis were the most common approaches used. Among the 24 studies based on other surveys, ten analyzed overall empowerment, while the others addressed sexual and reproductive health (4 studies), agriculture (3) and livestock (1), water and sanitation (2), nutrition (2), agency (1), and psychological empowerment (1). These measures were mainly based on data from single countries and factor analysis was the most frequently analytical method used. We observed a diversity of indicator definitions and domains and a lack of consensus in terms of what the proposed indicators measure.

**Conclusion:**

The proposed women's empowerment indicators represent an advance in the field of gender and development monitoring. However, the empowerment definitions used vary widely in concept and in the domains/dimensions considered, which, in turn influence or are influenced by the adopted methodologies. It remains a challenge to find a balance between the need for a measure suitable for comparisons across populations and over time and the incorporation of country-specific elements.

## 1. Introduction

Power relations that impede women from attaining healthy and fulfilling lives operate at many levels of society, from the most personal to the highly public (United Nations Development Programme, 1994). The limited opportunities to thrive that many women experience are seen as a result, among other elements, of their lack of power and influence in society, lack of choice and autonomy, work overburden, and discrimination. Along with gender equality, the empowerment of women is considered an effective way to fight against poverty, hunger, and disease, and to stimulate truly sustainable development (United Nations General Assembly, 2000).

In the academic literature, empowerment has been defined in many ways, often drawing on constructs of agency, choice, opportunities, resources, and power (Rowlands, 1998; Malhotra et al., 2002; Alsop and Heinsohn, 2005; Ibrahim and Alkire, 2007). The publications by Naila Kabeer are some of the most cited when defining women's empowerment in academic articles. She defines empowerment as a “*process of change by which those who have been denied the ability to make choices acquire such an ability”* (Kabeer, 1999, 2005). Accordingly, a woman to whom choice is denied is disempowered and a woman who has the possibility of making her choices may be powerful but not empowered if she has never been denied those choices (Kabeer, 2005). This definition views empowerment as a dynamic process that involves change over time, and that comprises the following inter-related dimensions: *resources or pre-conditions*, including access to, but also future claims to, material, human, and social resources; *agency or process*, the ability to define one's goals and act upon them, including the process of decision-making, negotiation, manipulation, etc.; and *achievements or outcomes*, understood as the wellbeing consequences of being empowered. All three are interconnected through an active process (Kabeer, 1999). In summary, the empowerment of an individual should be reflected in the ability to translate choices into action to finally achieve the desired outcomes (Alsop and Heinsohn, 2005). However, it is important to note that although being used to define a process, the term empowerment is more frequently used in the literature to refer to an observed status of a person or a group instead, which, in turn, could reflect the underlying empowerment process of change (Alsop and Heinsohn, 2005; Raj et al., 2021).

Women's empowerment is centered on a way of change that modifies the placement of those in a lesser position due to their gender to allow autonomy and self-determination and has been recognized as an essential part of the effort to promote development and as a goal in itself (Raj et al., 2017). Therefore, women's degree of empowerment is defined by gender and gender relations in society, which makes it highly specific in terms of culture and context.

With an increasing interest in monitoring progress, how to quantify women's empowerment has received a good deal of attention from international organizations and scholars, and quantitative measures have become increasingly common in the global development arena (Gram et al., 2017). Such measures are key for assessing levels of empowerment in countries as well as within social and geographic subgroups, and for exploring the impact of empowerment on health, wellbeing, and economic outcomes. However, women's empowerment is difficult to measure because the concept has diverse definitions and encompasses a broad spectrum of aspects of women's daily lives (Bishop and Bowman, 2014; Ewerling et al., 2017). Numerous metrics have been proposed, however, most of them face the inherent difficulties of measuring a process, which often leads to measures of status instead (Sharaunga et al., 2019). Also, despite the consensus on women's empowerment being a multidimensional construct, the dimensions themselves are far from consistent across conceptualizations and are also employed with different meanings and often used interchangeably.

To provide a better understanding of the current situation of women's empowerment measures, we undertook a scoping literature review aiming to summarize the indicators based on individual-level data and the methodologies used to derive them.

## 2. Material and methods

A literature search was conducted through PubMed, Scopus, Web of Science Core Collection, and Science Direct databases in June 2021 and updated in September 2022. Key terms related to women's empowerment were identified and combined using Boolean operators with no restrictions on language or publication year as presented in Table 1. Google and Google Scholar were used to identify relevant gray literature. We also manually searched publications on the websites of organizations undertaking research on women's empowerment: WHO Publications (https://www.who.int/publications); IRIS–Institutional Repository for Information Sharing (https://apps.who.int/iris/); UNICEF–Girls empowerment (https://www.unicef.org/topics/girls-empowerment); Naila Kabeer's website–Professor at the Gender Institute, London School of Economics and Political Science (http://nailakabeer.net/); Land Portal (https://landportal.org/); Center on Gender and Equity Health (https://gehweb.ucsd.edu); and EMERGE–Evidence-based Measures of Empowerment for Research on Gender Equality (https://emerge.ucsd.edu).

**Table 1:** Search strategy.

**PUBMED**
**(women empowerment) AND (“empowerment”[Title/Abstract]) OR (“decision making”[Title/Abstract]) OR (“bargaining”[Title/Abstract]) OR (“power”[Title/Abstract]) OR (“autonomy”[Title/Abstract]) OR (“agency”[Title/Abstract]) OR (“status”[Title/Abstract]) OR (“control”[Title/Abstract])**
**WEB OF SCIENCE, SCIENCE DIRECT, and SCOPUS**
**TITLE=((“empowerment” OR “decision making” OR “bargaining” OR “power” OR “autonomy“ OR ”agency“ OR ”status“ OR ”control“) OR ABSTRACT=(”empowerment“ OR ”decision making“ OR ”bargaining“ OR ”power“ OR “autonomy” OR “agency” OR “status” OR “control”) AND ALL FIELDS=(“women's empowerment” or “woman empowerment”))**

Publications describing the operationalization of a measure of women's empowerment as the main objective of the study and that relied upon individual-level data were eligible for our review. Documents that focused exclusively on empowerment at the workplace, specific professionals (such as nurses, midwives, caregivers, and sex workers), clinical environment, women in a situation of violence, with specific diseases or health conditions (such as cancer, epilepsy, HIV infections, postpartum depression) were excluded. There was no limit in terms of women's age or presence of children.

All the documents retrieved from the different databases were inserted into a reference manager software (Endnote^®^) and duplicates were removed automatically, followed by a manual revision. At the first stage of publication selection, titles and abstracts were independently screened by three reviewers (JC, MFM, GS). Studies for which inclusion was uncertain were left for full-text review. The publications selected in the previous step were reviewed in full text, and data of interest were extracted manually and independently documented in a structured extraction spreadsheet by each of the three reviewers. Reference lists of selected publications were screened for further relevant documents. Disagreements regarding whether a manuscript should be included in the review were resolved by consensus.

All analyses were based on previously published articles; therefore, no ethical approval or patient consent was required.

## 3. Results

**Figure 1:**
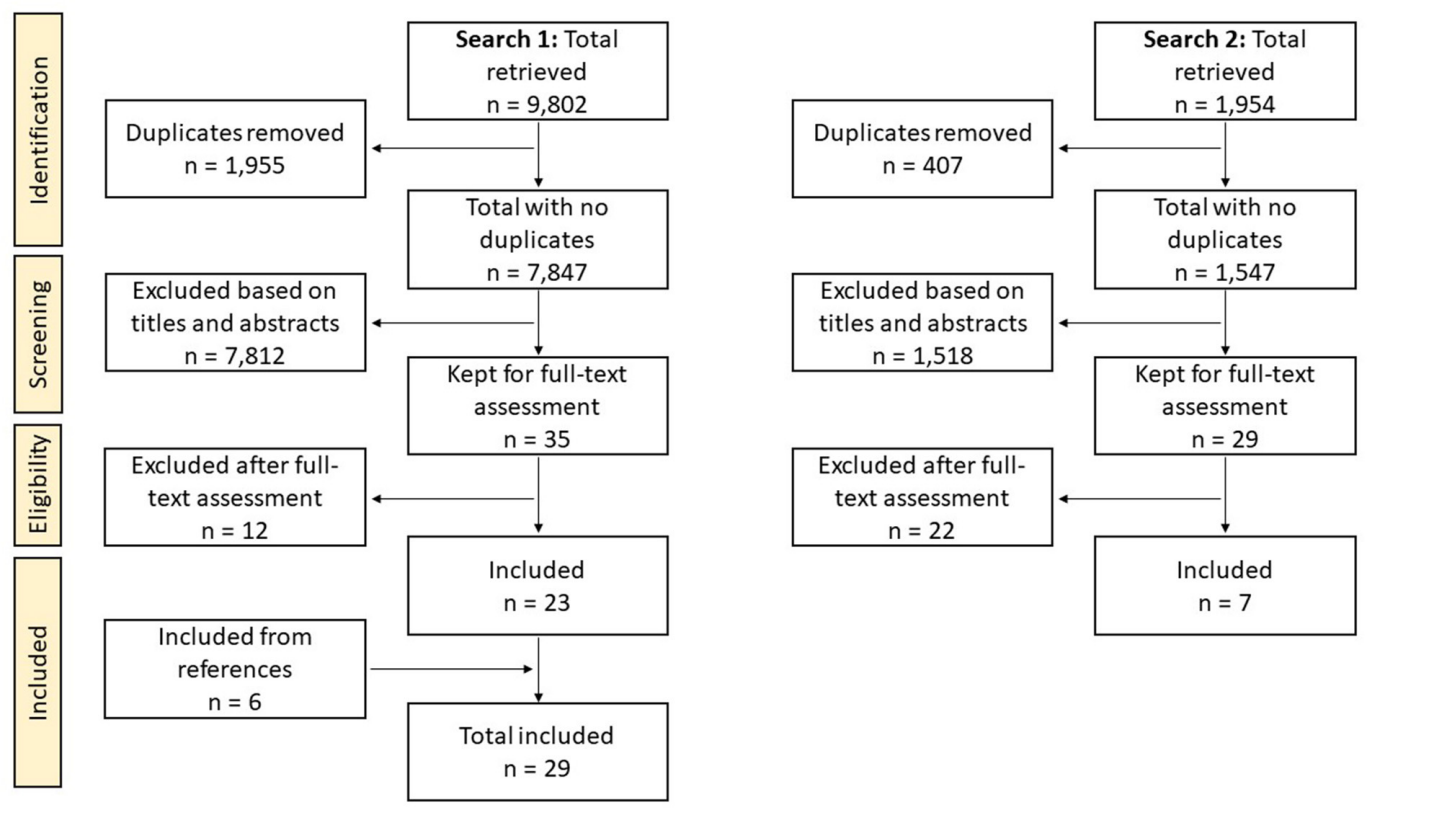
Flowchart of the study selection procedure.

A total of 9,802 publications were retrieved from the databases in the first search and 1,954 in the search update. After duplicates removal, titles and abstracts screening, and full-text assessment, we ended up with 36 publications to be included in this review as illustrated in Figure 1.

Out of these 36 publications proposing women's empowerment measures based on data collected at the individual level, 12 relied on Demographic and Health Surveys (DHS) and 24 on other surveys. All studies were published on or after 2004, 55% of them between 2019 and 2022. In most studies empowerment was conceptually defined as a process of change, however, in the end, the proposed indicators were mostly measures of status, most likely due to the cross-sectional nature of the data. Characteristics of the publications and the corresponding reference are presented in Table 2, with the main aspects highlighted in the next paragraphs. The measures are presented by type of survey (DHS or other surveys) and then described in more detail in the following sections.

**Table 2:** Characteristics of the publications proposing a women's empowerment measure.

**Characteristics**	**DHS-based surveys (*n =* 12)**	**Other surveys (*n =* 24)**
Publication type	• Peer-reviewed papers (Alkire et al., 2013; Phan, 2016; Ewerling et al., 2017, 2020; Miedema et al., 2018; Obayelu and Chime, 2020; Rettig et al., 2020; Soharwardi and Ahmad, 2020; Abbas et al., 2021; Annan et al., 2021; Mganga et al., 2021) • Report (Kenya National Bureau of Statistics, 2020)	• Peer-reviewed paper (Biswas and Kabir, 2004; Santillán et al., 2004; Varghese, 2011; Alkire et al., 2013; Bhattacharya and Banerjee, 2013; Upadhyay et al., 2014; Yount et al., 2016; Rafiey et al., 2018; Galiè et al., 2019; Hinson et al., 2019; Malapit et al., 2019; Narayanan et al., 2019; Sharaunga et al., 2019; Moreau et al., 2020; Dickin et al., 2021; Moubarak et al., 2021; Sharma and Das, 2021; Saha and Narayanan, 2022; Sinharoy et al., 2022) • Report (Lombardini et al., 2017; The Hunger Project, 2017; MEASURE Evaluation, 2020) • Other (e.g., discussion paper, working paper) (Williams, 2005; Malapit et al., 2017)
Empowerment areas	• Overall empowerment [All publications]	• Overall empowerment (Biswas and Kabir, 2004; Santillán et al., 2004; Williams, 2005; Varghese, 2011; Bhattacharya and Banerjee, 2013; Lombardini et al., 2017; The Hunger Project, 2017; Rafiey et al., 2018; Sharaunga et al., 2019; Sharma and Das, 2021) • Agency (Yount et al., 2016) • Sexual and Reproductive Health (Upadhyay et al., 2014; Hinson et al., 2019; MEASURE Evaluation, 2020; Moreau et al., 2020) • Nutrition (Narayanan et al., 2019; Saha and Narayanan, 2022) • Agriculture (Alkire et al., 2013; Malapit et al., 2017, 2019) and livestock (Galiè et al., 2019) • Water, sanitation, and hygiene (Dickin et al., 2021) or urban sanitation (Sinharoy et al., 2022) • Psychological (Moubarak et al., 2021)
Geography	• Low- and middle-income countries (Ewerling et al., 2020; Soharwardi and Ahmad, 2020) • African countries (Ewerling et al., 2017) • Sub-Saharan African countries (Asaolu et al., 2018; Annan et al., 2021) • East Africa (Miedema et al., 2018) • Asian countries (Phan, 2016) • Single countries: - Kenya (Kenya National Bureau of Statistics, 2020) - Nigeria (Obayelu and Chime, 2020; Rettig et al., 2020) - Pakistan (Abbas et al., 2021) - Tanzania (Mganga et al., 2021)	• South Asia and Sub-Saharan Africa (Sinharoy et al., 2022) • African countries - Kenya, Zambia, and Nigeria (MEASURE Evaluation, 2020) - Ethiopia, Uganda, and Nigeria (Moreau et al., 2020) - Benin, Burkina Faso, Ethiopia, Ghana, Malawi, Mozambique, and Senegal (The Hunger Project, 2017) • Multiple countries (two or more) from different regions - Bangladesh, Guatemala, and Uganda (Alkire et al., 2013) - Bangladesh, Burkina Faso, Ethiopia, Ghana, India, Kenya, Mali, Nepal, Tanzania (Malapit et al., 2019) - Bangladesh and Uganda (Malapit et al., 2017) • Single countries - Bangladesh (Biswas and Kabir, 2004; Williams, 2005) - Burkina Faso (Dickin et al., 2021) - Egypt (Yount et al., 2016) - India (Bhattacharya and Banerjee, 2013; Narayanan et al., 2019; Sharma and Das, 2021; Saha and Narayanan, 2022) - Iran (Rafiey et al., 2018) - Nepal (Hinson et al., 2019) - Oman (Varghese, 2011) - Saudi Arabia (Moubarak et al., 2021) - South Africa (Sharaunga et al., 2019) - Tanzania (Galiè et al., 2019) - Vietnam (Santillán et al., 2004) - United States (Upadhyay et al., 2014) • Methodological only (for any setting of interest) (Lombardini et al., 2017)
Population	• Married women aged 15–49 years (Ewerling et al., 2017, 2020; Asaolu et al., 2018; Miedema et al., 2018; Soharwardi and Ahmad, 2020; Abbas et al., 2021; Mganga et al., 2021) • Women aged 15–49, currently married, currently working or worked in the past 12 months, earn cash or cash and kind, sexually active and fecund (target sample by design) (Phan, 2016) • Married and unmarried women aged 15–49 years (Kenya National Bureau of Statistics, 2020) • Rural women (Obayelu and Chime, 2020) • Couples (Annan et al., 2021)	• Women aged 15–49 years (Moreau et al., 2020) • Married women (22–52 years) from rural communes and their husbands (Santillán et al., 2004) • Married/partnered women aged 15–49 years (MEASURE Evaluation, 2020) or 16–49 years (Yount et al., 2016) • Women of reproductive age (18–49 years) (Bhattacharya and Banerjee, 2013) • Women from randomly selected households (Rafiey et al., 2018) • Self-identified primary male and female adult decision-makers aged 18 and older (Alkire et al., 2013; Malapit et al., 2017; Dickin et al., 2021) • Women aged 15 years or over (Williams, 2005; Galiè et al., 2019) • Adult women aged 18 years or more (Sinharoy et al., 2022) • Beneficiaries of the interventions in each site (as many of the projects are targeted at women, the author assumes that the eligible participant is a woman) (Malapit et al., 2019) • Women who had at least one child under the age of seven and who were previously surveyed, their husbands and mothers-in-law (Narayanan et al., 2019) • Young mothers with children below the age of five, male spouses and mother-in-law, and elder women above the age of 70 (Saha and Narayanan, 2022) • Women aged 15–60 years attending family-planning or abortion facilities (Upadhyay et al., 2014) • Women aged 20–35 years who had been married for at least six months and who currently live with their partners (Hinson et al., 2019) • Rural women from randomly selected households (Sharaunga et al., 2019) or villages (Sharma and Das, 2021) • Women from a convenience sample of households (Varghese, 2011) • Saudi women from different cultures (Moubarak et al., 2021) • Not informed (Biswas and Kabir, 2004; Lombardini et al., 2017; The Hunger Project, 2017)
Analytical approach	• Exploratory/confirmatory factor analysis (Phan, 2016; Asaolu et al., 2018; Miedema et al., 2018; Kenya National Bureau of Statistics, 2020; Mganga et al., 2021) • Principal components analysis (Ewerling et al., 2017, 2020; Soharwardi and Ahmad, 2020) • Mean of all categories scores (Rettig et al., 2020) • Alkire-Foster method (Obayelu and Chime, 2020) • Binary composite indicator (Abbas et al., 2021) • Multiple category indicator (Annan et al., 2021)	• Exploratory/confirmatory factor analysis (Williams, 2005; Upadhyay et al., 2014; Yount et al., 2016; Rafiey et al., 2018; MEASURE Evaluation, 2020; Moubarak et al., 2021; Sharma and Das, 2021) • Exploratory/confirmatory factor analysis and item response theory (Sinharoy et al., 2022) • Principal component analysis and exploratory factor analysis (Moreau et al., 2020) • rincipal component analysis (Sharaunga et al., 2019) • Alkire-Foster method (Alkire et al., 2013; Malapit et al., 2017, 2019; Narayanan et al., 2019; Dickin et al., 2021; Saha and Narayanan, 2022) • Multiple-indicator-multiple-cause model (Bhattacharya and Banerjee, 2013; Yount et al., 2016) • Structural equation model (Bhattacharya and Banerjee, 2013) • Construction method used for the Human Development Index and average of the three dimensions (Varghese, 2011) • Scaling system for quantification of the responses of each sub-indicator (question/variable) and percentage for each indicator (domains); weighted or unweighted mean for the composite index (Biswas and Kabir, 2004) • Sum of the domain-specific categorical variables resulting in a single continuous variable/score (Santillán et al., 2004; Hinson et al., 2019) • Average of indicators and dimensions (Lombardini et al., 2017)

Eleven out of the 12 studies based on DHS data were published as peer-reviewed papers and one as a report. All of them proposed measures of women's empowerment with a broad perspective, henceforth called *overall empowerment*, as opposed to focusing on a specific domain (such as decision-making and agency) or a woman's life area (such as agriculture and nutrition). The analyses included populations from sets of countries from specific regions such as Asia or Africa, or low- and middle-income countries (LMICs) generally. Five studies were based on data from single countries (Kenya, Pakistan, Tanzania, and two from Nigeria). Given the specific survey design, DHS-based studies analyzed the empowerment of women aged 15–49 years. One study focused on rural women only, two included unmarried women in the analysis and one analyzed couple's responses. Factor analysis (exploratory, confirmatory, or both) was used in four studies, principal component analysis (PCA) was used in three while the remaining two used either the Alkire-Foster approach, an axiomatic and counting-based approach designed originally for measuring multidimensional poverty (Alkire and Foster, 2011), or sum of scores. Three studies did not rely on statistical approaches to define the measure and used the sum of scores, a binary indicator of empowerment, or a categorical variable of responses' agreement. The publications in each category can be identified in Table 2.

Regarding the 24 studies based on data from non-DHS surveys, we identified 19 peer-reviewed papers, three reports, and two papers that were not published in peer-reviewed academic journals. Here, the studies typically covered a single country, or sets of selected countries from different regions, usually Africa and South Asia. One publication proposed the methodology for quantitatively measuring women's empowerment in Bangladesh without using data and another presented the steps to calculate an empowerment measurement that could be employed in different contexts with no specific setting under analysis. Ten studies in this group analyzed overall empowerment, while the remaining looked at specific areas of women's life: sexual and reproductive health (4 studies), agriculture (Alsop and Heinsohn, 2005) and livestock (United Nations Development Programme, 1994), water and sanitation (United Nations General Assembly, 2000), nutrition (United Nations General Assembly, 2000), agency (United Nations Development Programme, 1994), and psychological empowerment (United Nations Development Programme, 1994). The population under study varied widely in this group of publications: some studies included both women and men, while others were based on data from ever-married women in different age groups or women from rural areas only. The most used analytical methods were exploratory/confirmatory factor analysis (9 studies, one of which also used the Item Response Theory), followed by the Alkire-Foster approach (Rowlands, 1998) and the Multiple-Indicator-Multiple-Cause model (United Nations General Assembly, 2000). The other methods used and specific characteristics of each publication can be identified in Table 2.

### 3.1. Measures based on demographic and health survey data

In general, publications describing DHS-based measures were consistent in recognizing the multidimensional nature of women's empowerment. However, these constitutive elements of empowerment received different names, such as components (Phan, 2016), categories (Rettig et al., 2020), domains (Ewerling et al., 2017, 2020; Miedema et al., 2018; Kenya National Bureau of Statistics, 2020; Obayelu and Chime, 2020), dimensions (Soharwardi and Ahmad, 2020), or both dimensions and domains (Asaolu et al., 2018). Also, there was no consensus on a hierarchy of dimensions and domains in terms of having a comparable structure across studies.

The methodologies used by the studies' authors to define these elements were based on literature review, theoretical background, or conceptual framework (Phan, 2016; Asaolu et al., 2018; Miedema et al., 2018; Kenya National Bureau of Statistics, 2020; Soharwardi and Ahmad, 2020); factor analysis (Phan, 2016; Ewerling et al., 2017; Asaolu et al., 2018; Miedema et al., 2018; Kenya National Bureau of Statistics, 2020; Soharwardi and Ahmad, 2020); consultation of experts or organizations (Ewerling et al., 2020); and adaptation from other indices (Ewerling et al., 2020; Obayelu and Chime, 2020). Hereafter we refer to these elements as domains.

Two sets of DHS questions were used to define domains in several of the publications. The first is the set of decision-making questions, named decision-making in seven studies and agency/autonomy in another. The second is the set of questions on whether the woman believes a husband is justified in beating the wife in specific circumstances. Five studies used this set for a domain that received names such as attitude to violence/perception of violence/violence against women/attitude toward wife-beating. A further two domains were based on the same set of variables—human and social resources/human and social assets/social independence/socio-cultural in four publications and labor force participation/women's work status/employment in three publications.

In Table 3, we list all 56 variables from DHS questionnaires that were included in at least one empowerment measure identified by this review and the corresponding domain to which they were assigned, as named by the study authors.

**Table 3:** Variables used in DHS-based women's empowerment measures and their corresponding domains.

**Question**	**Times used**	**Asaolu et al. (2018)**	**Ewerling et al. (2017)**	**Ewerling et al. (2020)**	**Kenya National Bureau of Statistics (2020)**	**Miedema et al. (2018)**	**Obayelu and Chime (2020)**	**Phan (2016)**	**Rettig et al. (2020)**	**Soharwardi and Ahmad (2020)**	**Abbas et al. (2021)**	**Mganga et al. (2021)**	**Annan et al. (2021)**
		**19 SSA countries**	**33 African countries**	**62 LMICs**	**Kenya**	**5 East African countries**	**Rural Nigeria**	**4 South Asian countries**	**Nigeria**	**38 LMICs**	**Pakistan**	**Tanzania**	**23 SSA countries**
Frequency read newspaper or magazine	5		Social independence	Social independence	Human and social resources					Awareness		Social independence	
Frequency of watching TV	3				Human and social resources					Awareness		Social independence	
Frequency of listening to radio	2				Human and social resources					Awareness			
Age at first birth	6	Socio-cultural: life course	Social independence	Social independence		Human and social assets			Reproductive healthcare			Age at critical life events	
Age at first cohabitation	6	Socio-cultural: life course	Social independence	Social independence		Human and social assets			Violence against women			Age at critical life events	
Age difference (husband–woman)	3		Social independence	Social independence		Human and social assets							
Beating is justified if wife goes out without telling husband	9	Socio-cultural: attitude toward violence	Attitude to violence	Attitude to violence	Socio-cultural	Gendered beliefs	Agency		Violence against women	Self-esteem		Attitudes toward violence	
Beating is justified if wife neglects the children	9	Socio-cultural: attitude toward violence	Attitude to violence	Attitude to violence	Socio-cultural	Gendered beliefs	Agency		Violence against women	Self-esteem		Attitudes toward violence	
Beating is justified if wife argues with husband	9	Socio-cultural: attitude toward violence	Attitude to violence	Attitude to violence	Socio-cultural	Gendered beliefs	Agency		Violence against women	Self-esteem		Attitudes toward violence	
Beating is justified if wife refuses to have sex with husband	9	Socio-cultural: attitude toward violence	Attitude to violence	Attitude to violence	Socio-cultural	Gendered beliefs	Agency		Violence against women	Self-esteem		Attitudes toward violence	
Beating is justified if wife burns the food	9	Socio-cultural: attitude toward violence	Attitude to violence	Attitude to violence	Socio-cultural	Gendered beliefs	Agency		Violence against women	Self-esteem		Attitudes toward violence	
Beating is justified if wife has sex outside of marriage	1					Gendered beliefs^#^							
Who usually decides on respondent's health care?	11	Socio-cultural: household decision-making	Decision-making	Decision-making	Familial/ interpersonal (household and sexual/ reproductive decision-making) ^*^	Decision-making	Agency^&^	Decision-making	Decision-making	Decision-making	Decision-making (health)	Decision making	
Who usually decides on large household purchases?	11	Socio-cultural: household decision-making	Decision-making	Decision-making	Familial/ interpersonal^*^	Decision-making	Agency	Decision-making	Decision-making	Decision-making	Decision-making (economic)	Decision making	No domain assigned
person who usually decides on household purchases for daily needs (now dropped)	2					Decision-making			Decision-making				
Who usually decides on visits to family or relatives?	10	Socio-cultural: household decision-making	Decision-making	Decision-making	Familial/ interpersonal^*^	Decision-making		Decision-making	Decision-making	Decision-making	Decision-making (mobility)	Decision making	
Who usually decides how your (husband's/partner's) earnings will be used?	7				Familial/ interpersonal^*^	Decision-making		Decision-making	Decision-making^$^	Decision-making	Decision-making (economic)	Decision making	
Who usually decides food to be cooked each day? (now dropped)	1								Decision-making				
Who usually decides how respondent's earnings will be used?	1											Decision making	
Woman's education	8	Education: highest educational level	Social independence	Social independence	Human and social resources	Human and social assets		Education	Education			Social independence	
Education difference (husband - woman)	4	Education: spousal/partner difference in educational level	Social independence	Social independence		Human and social assets							
Women's literacy	5	Education: literacy				Human and social assets^+^	Resources	Education	Education				
Ownership of house	5	Socio-cultural: land or home ownership			Economic^*^		Leadership				Ownership	Property ownership	
Ownership of land	5	Socio-cultural: land or home ownership			Economic^*^		Leadership				Ownership	Property ownership	
Ownership of a mobile telephone	1											Social independence	
Would you say that the money that you earn is more than what your (husband/partner) earns, less than what he earns, or about the same?	3	Economic: labor force participation				Human and social assets		Labor force participation					
Demand for family planning satisfied	3				Familial/ interpersonal^*^			Family planning	Access to contraceptives				
When you are sick and want to get medical advice or treatment, is each of the following a big problem or not a big problem: a) getting permission to go to the doctor?	4	Health: access to healthcare			Familial/interpersonal^*^			Decision-making				Access to healthcare	
b) getting money needed for advice or treatment?	4	Health: access to healthcare						Decision-making		Self-confidence		Access to healthcare	
c) distance to health facility	2	Health: access to healthcare										Access to healthcare	
d) not wanting to go alone	4	Health: access to healthcare						Decision-making		Self-confidence		Access to healthcare	
Is a wife justified in refusing to have sex with her husband when she knows he has sex with other women? (HIV/AIDS section)	1					Gendered beliefs							
If a wife knows her husband has a disease that she can get during sexual intercourse, is she justified in asking that they use a condom when they have sex? (HIV/AIDS section)	1					Gendered beliefs							
Can you say no to your (husband/partner) if you do not want to have sexual intercourse? (HIV/AIDS section)	2	Health: sex negotiation			Familial/ interpersonal^*^								
Could you ask your (husband/partner) to use a condom if you wanted him to? (HIV/AIDS section)	2	Health: sex negotiation			Familial/ interpersonal^*^								
Respondent's occupation	3						Income	Labor force participation		Work status			
Respondent works for family, others, self	2	Economic: labor force participation						Labor force participation					
Type of earning from respondent's work/Work for cash and/or in-kind	4	Economic: labor force participation				Human and social assets		Labor force participation				Social independence	
Seasonality of respondent's occupation	3	Economic: labor force participation			Economic			Labor force participation					
Currently working	1									Work status			
Respondent worked in the past 12 months	3		Social independence					Labor force participation	Employment				
Time spent in sourcing water	1						Time/workload						
Knowledge of modern contraception	2				Human and social resources^*^			Family planning					
Exposure to family planning information	1				Human and social resources^*^								
Knowledge about access to contraception	1				Human and social resources								
Comprehensive knowledge about HIV/AIDS	1				Human and social resources								
Woman thinks female genital mutilation (FGM) should be stopped	1				Socio-cultural^*^								
Age at first sex	1					Human and social assets							
First sex at marriage	1					Human and social assets							
Discuss family planning with health worker	1						Leadership						
Heard about family planning on the radio	2							Family planning		Awareness			
Heard about family planning on TV	2							Family planning		Awareness			
Heard about family planning in newspapers	2							Family planning		Awareness			
Prenatal care visit for the most recent child	2						Resources		Reproductive healthcare				
Was respondent's most recent child delivered in a professional setting?	1								Reproductive healthcare				
Type of cooking fuel	1						Time/workload						
**Total of variables used in the empowerment measure**		**25**	**15**	**14**	**25**	**23**	**15**	**20**	**19**	**19**	**6**	**23**	**1**

KNBS, Kenya National Bureau of Statistics; SSA, sub-Saharan African countries; LMIC, low- and middle-income countries. ^*^Questions included for married women only. ^#^Question included for Rwanda only. ^$^Information presented in the text refers to the question “Does respondent have a say in deciding what to do with money?”, therefore it is not possible to affirm if it refers to respondent's money or partner's money. ^&^Information presented in the text refers to the “Decision on visit to community facility.” ^+^A dichotomous variable of literacy was originally included in EFA models but was dropped due to multicollinearity with the continuous schooling attainment.

The number of variables in each study ranged from one to 25. The variables most consistently used were those related to who primarily decides on the woman's own health care (nine studies), large household purchases (nine), and visits to family or relatives (eight). Next are the five questions related to whether the woman agrees that a husband is justified in beating his wife in specific situations (all questions in 8 studies). Then comes women's education included in seven studies. Used in five of the studies are age at first birth, age at first marriage/cohabitation, participation in decisions related to partner's earnings, and woman's literacy. The questions on whether the woman can refuse to have sex with her husband or can ask him to use a condom under specific situations are in the HIV/AIDS module that is applied only in specific countries where HIV is endemic and cannot be included in measures to be used in a wider selection of countries, therefore, only two studies included this set of information (Asaolu et al., 2018; Kenya National Bureau of Statistics, 2020).

Next, we present a summary of the methods employed in each study. One publication started the construction of the measure with a hierarchical attribution of dimensions and domains (Asaolu et al., 2018). From theory, the studies' authors identified and selected four empowerment dimensions: economic, socio-cultural, education, and health. Then the variables selected from the survey questionnaires were organized into ten domains. The economic dimension, for instance, had only one domain—labor force participation. In the socio-cultural dimension, on the other hand, the variables were organized in four domains: household decision-making, attitude toward violence, life course indicator, and land or home ownership. The results of the exploratory and confirmatory factor analyses identified four dimensions that the study's authors then call factors: education, attitude toward violence, labor force participation, and access to healthcare (Asaolu et al., 2018).

A more pragmatic approach was taken by Ewerling, Lynch (Ewerling et al., 2017) to create the survey-based women's empowerment index (SWPER) for African countries, where, after selecting suitable variables based on the concept of women's empowerment, PCA was used to understand how those variables were organized into empowerment domains. Here, three domains were found and named social independence, attitude to violence, and decision-making (Ewerling et al., 2017). In the SWPER updated version, which expands the original indicator developed for African countries to the whole of LMICs, these authors adapted the content by excluding women's working status and recategorizing the decision-making-related items (Ewerling et al., 2020). A similar approach was used by Soharwardi and Ahmad (2020), who used PCA and 19 indicators selected with a basis on a theoretical background to define five broad dimensions of empowerment: women's work status, awareness, participation in decision-making, self-esteem, and self-confidence (Soharwardi and Ahmad, 2020). This study's authors also used multiple linear regression models to evaluate the relationship between women's empowerment and socio-economic and demographic characteristics of households and household members (here are included, among other variables, women's education, age at first birth, and age difference with husband).

Other publications started with a conceptual model but grouped the variables and named the resulting domains of empowerment based on a more data-driven approach. Miedema et al. (2018), for instance, started with 24 variables grouped into three dimensions (human and social assets, gender beliefs and attitudes, and household decision-making). After running factor analysis, they ended up with three latent factors that kept the original structure but were based on a smaller set of 12 variables (Miedema et al., 2018). A similar approach was used to assess individual and country-level changes in empowerment in Tanzania over time. The study selected the first set of 27 variables from previous literature and then employed factor analysis to identify the 23 relevant ones and defined six domains based on the factor loadings: attitudes toward violence, decision-making, social independence, age at critical life events, access to healthcare, and property ownership (Mganga et al., 2021).

Orthogonal and oblique factor models were used by Phan (2016) to calculate women's empowerment scores based on 12 variables covering four aspects (or domains) of women's empowerment (labor force participation, household decision-making, family planning, and education). The analyses were performed separately for each of the four countries included in the study (Cambodia, Indonesia, the Philippines, and Timor-Leste). The four items of labor force were consistently identified in the first factor and education items in the second factor. The third factor consisted of three decision-making items in all countries but Timor-Leste while family planning did not appear to be one relevant factor in these analyses.

When constructing the Women's Empowerment Index (WEI) for Kenya, based on aspects of women's empowerment relevant to the country's context, these authors relied upon a review of national and international policy and legal documents and consultations with national stakeholders and development partners (Kenya National Bureau of Statistics, 2020). Only indicators that fulfilled the criteria of great relevance, correspondence with the literature on the topic and with Kabeer's conceptual framework, high variance, and low percentage of missing values were kept in the analysis. The empirical analysis was carried out separately for women in union and women not in union, comprising different sets of variables. The exploratory and confirmatory factor analysis models identified 18 indicators in five domains of empowerment for partnered women (economic, human and social resources, household and decision-making, control over sexual relations, and attitudes toward wife-beating) and 11 indicators distributed in three domains for unpartnered women (economic, human and social resources, and attitudes toward wife-beating) (Kenya National Bureau of Statistics, 2020). To construct the WEI, equal weights were assigned to the domains, and the weight of the domain was distributed equally to its indicators. Therefore, a woman was considered empowered if reached at least 80 percent of the total weighted indicators.

Obayelu and Chime (2020) defined the five domains of the Women's Empowerment Index as agency, resources, income, leadership, and time/workload based on the index previously proposed by The Hunger Project, which in turn has the Women's Empowerment in Agriculture Index (WEAI) as reference (Alkire et al., 2013; The Hunger Project, 2017). Also, the index has the gender parity index component to compare women's and men's achievements. To identify the areas that contribute most to women's disempowerment, it was decomposed by domain and indicators using the Alkire and Foster (2007) methodology. Socioeconomic characteristics that influence women's empowerment were also explored, including women's age, education, marital status; household wealth; and partner's age and education level.

The Female Empowerment Index (FEMI) was developed to assess women's empowerment over time in Nigeria. It was computed based on 19 indicators as the resulting index is expressed as the average proportion (therefore, ranging from 0 to 1) of positive outcomes in six categories: violence against women, employment, education, reproductive healthcare, decision-making, and access to contraceptives (Rettig et al., 2020). The sample includes both married and unmarried women and the measure was estimated for each of the 36 Nigerian states. In addition to the FEMI values, for some categories (such as employment and education) it was possible to estimate women's achievement relative to men's.

Abbas et al. (2021) operationalized empowerment as a binary variable resulting from the combination of two independent domains: decision-making, a dichotomous indicator coded 0 for no involvement at all in any decision and coded 1 for any kind of involvement, either alone or jointly; and ownership of property, coded as 1 when the woman owns a house or land alone or jointly and 0 when she owns neither a house nor a land. Women's empowerment was then recoded as not empowered when the woman was not at all involved in household decision-making and did not possess a house or land and empowered otherwise (Abbas et al., 2021). This variable was used as the dependent variable in a regression analysis aiming to identify determinants of women's empowerment.

A different approach was adopted by Annan et al. (2021), who used a sample of married couples in 23 Sub-Saharan African countries where both spouses answered the question “who usually makes decisions about making major household purchases?” to evaluate their agreement. These authors operationalized the measure in four categories: the woman attributes more decision-making power to herself than her husband does; the husband attributes more decision-making power to the woman than she does to herself; both agree the woman is the main decision-maker or that decision-making is joint; and both agree that husband is the main decisionmaker (Annan et al., 2021). The relationship between this indicator and other proxies of women's empowerment as well as its impact on women's and children's wellbeing outcomes were assessed using regression models.

### 3.2. Measures based on other surveys

The 24 empowerment measures that were developed based on data from surveys other than DHS varied in their objectives, empowerment dimensions and domains, indicators, data collection procedures, samples, and geographies. In contrast with the DHS-based ones, which were about overall empowerment, some of the measures presented in this section addressed a single empowerment dimension (as agency), specific areas related to women's lives (such as sexual and reproductive health and nutrition), or aspects related to the society/community (such as agriculture, or water and sanitation). A summary of study characteristics related to empowerment domains, measures, and data sources is presented in Table 4.

**Table 4:** Characteristics of measures based on survey data other than DHS.

**Measure**	**Empowerment area**	**Purpose**	**Domains**	**Measures/Analytical approach**	**Data sources**
Women's Empowerment in Agriculture Index (Alkire et al., 2013)	Agriculture	Measure the empowerment, agency, and inclusion of women in the agricultural sector and can be adapted to measure empowerment of women in rural areas more generally, whether they are farmers, agricultural or non-agricultural wage workers, or engaged in non-farm businesses.	05 domains (1) Decisions about agricultural production (2) Access to and decision-making power about productive resources (3) Control of use of income (4) Leadership in the community (5) Time allocation	Multidimensional index reported at the country or regional level, based on individual-level data, represents the percentage of women who are empowered (i.e., achieve adequate4 achievements in 80% of the weighted indicators or more) based on the Alkire–Foster methodology. WEAI = five domains of empowerment (90%) + gender parity index (GPI, reflects intra-household inequality, 10%).	Pilot survey with questionnaires, administered to the primary male and female respondent in each household in Feed the Future zones of influence in Bangladesh, Guatemala, and Uganda.
Empowerment in Water, Sanitation and Hygiene Index (EWI) (Dickin et al., 2021)	Water, Sanitation, and Hygiene	Measure agency, participation, and empowerment in the water and sanitation sector.	03 levels (1) Individual (2) Household (3) Societal - Community - Local WASH institutions and authorities	Empowered individuals are those achieving at least 75% of the indicators (threshold used in the project-level WEAI). The scores for men and women within the household are also compared to create an intra-household parity index (IHPI).	Individual-level survey data collected from male and female respondents from the same household (pilot in rural and peri-urban communities Banfora, Burkina Faso).
Women's Empowerment in Livestock Index (WELI) (Galiè et al., 2019)	Livestock	Assess the empowerment of women in the livestock sector	03 dimensions (1) Decisions about agricultural production (2) Decisions related to nutrition (3) Access to and control over resources (4) Control and use of income (5) Access to and control of opportunities (6) Workload and control over own time	Analogous to standard methods for computing the WEAI, with each dimension weighted as 1/6 and the summation produces the WELI score (ranges from 0 for the least empowered to 1 for the most empowered women)	Piloted in four districts of the “More Milk in Tanzania” project; survey conducted among women living in the households that were monitored regularly. for the project.
Measures of reproductive decision-making agency (Hinson et al., 2019)	Sexual and reproductive health	Develop and test measures that capture women's decision-making agency across multiple domains of reproductive health and capture core components of empowerment in the decision-making process by including elements of voice, power, and choice.	03 domains of reproductive decision making (1) Agency around when to have children (2) Agency around whether to use (3) Agency around which method of contraception	For each domain, a three-category variable indicating low, medium, or high agency was constructed, based on the four core questions. Next, the three domain-specific measures were combined into a single measure by constructing an additive scale from the three categorical variables. This resulted in a single continuous variable with values ranging from three to nine [women scoring three or four = low; five, six, or seve*n =* medium; eight or nine = high agency].	Primary data collection in Morang and Kaski Districts in Nepal
Project-level Women's Empowerment in Agriculture Index (pro-WEAI) (Malapit et al., 2019)	Agriculture	Adapts the WEAI for use as a metric for measuring the impact of agriculture development projects on women's empowerment, as well as a diagnostic tool for tailoring such programs to specific settings.	03 domains (1) Intrinsic agency (power within) (2) Instrumental agency (power to) (3) Collective agency (power with)	Uses the Alkire-Foster method. The index is comprised of the three domains of empowerment (3DE, 90%) and the GPI (10%). The empowerment score is the weighted average of adequacy in the 12 indicators (a score of at least 75% or considered adequate in at least nine indicators is classified as empowered).	Five agricultural development projects in the Gender, Agriculture, and Assets Project, phase 2 (GAAP2) portfolio that had explicit women's empowerment goals: Bangladesh, Burkina Faso, Ethiopia, Ghana, India, Kenya, Mali, Nepal, Tanzania
Reproductive Empowerment Scale (MEASURE Evaluation, 2020)	Sexual and reproductive health	Strengthen and standardize a measurement of reproductive empowerment among women in sub-Saharan Africa that can be incorporated in survey instruments	05 subscales (1) Women's communication with healthcare providers (2) Communication with partners (3) Decision-making (4) Social support (5) Social norms on issues related to women's reproductive health and fertility	The scale and/or each subscale should be scored by summing the numerical responses to each item (whole numbers from one to four) and then dividing the total score by the number of items in the subscale(s).	The scale was drafted based on a literature review and focus group discussions in Zambia; a draft scale was developed and then tested and refined through cognitive interviews with women in Kenya; and it was quantitatively validated within a broader family planning and reproductive health survey in Nigeria.
Women's and Girl's Empowerment–Sexual Reproductive Health (WGE-SRH) (Moreau et al., 2020)	Sexual and reproductive health	Evaluate women's motivations for choosing to have sex, use contraceptives, or become pregnant, and the constraints on their making these choices, across diverse Sub-Saharan African contexts.	02 domains for the three outcomes (sex, contraceptive use, and pregnancy) (1) Existence of choice (constraints or motivations surrounding preferences) (2) Exercise of choice (ways in which women and girls sought to implement these preferences through their decision making and negotiation tactics)	Psychometric properties were explored to identify cross-site constructs, and logistic regression was used to assess the construct validity of each dimension. Summary scores for each domain and outcome were calculated by averaging scores for relevant items as well as three outcome-specific scores by adding the relevant summary scores of the domains. Divided into tertiles for ease interpretation and application.	Data from Performance Monitoring for Action (PMA) project, formerly Performance Monitoring and Accountability 2020 (PMA2020) collected in 2017–2018 in urban and rural areas of Ethiopia, Uganda and two sites in Nigeria
Women's Empowerment in Nutrition Index (Narayanan et al., 2019)	Nutrition	Measure women's empowerment in the realm of nutrition, defined as the process by which individuals acquire the capacity to be well fed and healthy, in a context where this capacity was previously denied to them, but also to be predictive of nutritional status	03 dimensions (A) Knowledge (B) Material and social resources (C) Agency and autonomy 03 domains (1) Food (2) Health (includes fertility as subset of health, relevant only to women aged 15–49) (3) Institutions	A count of the number of domain-dimensions in which an individual is empowered (scores less than 0.5) is divided by the total number of domain-dimensions (7 or 10) to obtain WENI, which ranges from 0 to 1 (from completely nutritionally disempowered to fully empowered)	Fit-for-purpose survey in two states of India (Odisha and Bihar) that resamples women from 26 villages who were previously surveyed under the 2014–2016 Systematic for Promoting Appropriate National Dynamism for Agriculture and Nutrition (SPANDAN) project.
Women's Empowerment Index (Lombardini et al., 2017)	Overall empowerment	Design a measurement tool that could be used in efforts to establish causality when integrated within impact evaluation designs, while providing a numerical value for empowerment that could be generalized to the entire population under analysis	03 levels of change and 13 indicators (United Nations Development (United Nations Development Programme, 1994) Personal 1.1 Self-confidence 1.2 Individual knowledge 1.3 Opinions and attitudes on women's economic roles 1.4 Non-acceptance of gender-based violence 1.5 Personal autonomy 1.6 Individual capability (2) Relational 2.1 influencing and community 2.2 Control over household assets 2.3 Involvement in household decision making 2.4 Independent income 2.5 Experience of gender-based violence 2.6 Control over time (3) Environmental 3.1 Access to services and resources 3.2 Ability to influence at political level	The index represents the proportion of characteristics in which women score positively (meaning that they reach the defined cut-off points) across the indicators. The final index has a value ranging between 0 and 1 (less to more empowered),	Uses as an example the 2015/16 Effectiveness Review conducted in Armenia
Persian-version tool for measuring women's empowerment (Rafiey et al., 2018)	Overall empowerment	Develop and validate a women's empowerment questionnaire suitable for Iran.	04 subscales (1) Psychological empowerment (2) Decision-making empowerment (3) Social empowerment (4) Gender empowerment	The exploratory factor analysis revealed that 18 items of the model, scaled on a five-point Likert scale format, loaded on the 4 factors.	Questionnaire applied to women living in three suburban regions of the city of Dezful, Khuzestan province, Iran
Indicators to assess women's empowerment in Vietnam (Santillán et al., 2004)	Overall empowerment	Develop culturally appropriate indicators of women's empowerment, specific to the Vietnamese context	02 sets of domains (1) Women's social and economic roles 1. Production, 2. Housework, 3. Family expenditures, 4. Relations with family 5. Community participation 6. Rights of husbands and wives (2) Reproductive health 1. Childbearing 2. Contraception 3. Sexual communication and negotiation 4. Pregnancy 5. Appraisal of health services 6. Reproductive tract infections 7. Reproductive health roles and rights, including domestic violence and infidelity	A score of one was given if there was little or no evidence that the woman was empowered in a particular aspect, two if the evidence suggested that she was somewhat empowered, and three if she was considerably empowered. Each woman received a score for each domain and also received an overall score for empowerment in the socio-economic sphere and for empowerment in reproductive health.	Questionnaire applied to women from four rural communes in two provinces, Ninh Binh and Thanh Hoa, northern Vietnam
Measurement of Women's Empowerment (Sharaunga et al., 2019)	Overall empowerment	Develop an instrument to measure attitudes regarding women's empowerment, which can develop better understanding and future interventions to counter these negative attitudes for better social and health care, including mental and physical health.	04 levels/dimensions (1) Economic empowerment–economic agency–human capital–financial capital–vocational skills–physical asset (2) Social empowerment–social agency–social capital–informational asset (3) Empowerment in agriculture–crop management skills–farm financial management–water use security–women's socio-cultural hindrances to agriculture–animal production skills–weed and pest management skills (4) Civic empowerment–legal resource–civic agency–knowledge of rights–psychological resource–political resource	Used principal component analysis to generate factor scores at each dimension of empowerment as the better approach to quantitatively measure women's empowerment	Questionnaire applied to women living in randomly selected households in rural Msinga, South Africa
Custom Women's Empowerment Index (WEI) (The Hunger Project, 2017)	Overall empowerment	Measure progress in the multi-dimensional aspects of women's empowerment by aggregating results across domains	05 domains (1) Agency (2) Income (3) Leadership (4) Resources (5) Time	Composed by the women's achievement ratio (WAR, 60%) and the gender parity ratio (GPR, 40% of the score) and reported at the community level; higher value of WEI indicates greater empowerment; scores up to 100 points. There are five-equally weighted domains, each containing two to three indicators, that in turn, are given target thresholds of achievement.	Collected as part of a regular randomized household survey
Measurement of Women's Agency in Egypt (Yount et al., 2016)	Agency	Measure women's agency in the Arab Middle East, applying factor analysis to explore and test its factor structure	03 dimensions (1) Influence in family (economic) decisions [decision-making] (2) Freedom of movement (3) Attitudes regarding violence against wives	Factor loadings used to identify structure of women's agency; also tested differential item functioning by women's age at first marriage using multiple indicator multiple cause structural equations models).	Egypt Labor Market Panel Survey
Reproductive Autonomy Scale (Upadhyay et al., 2014)	Sexual and reproductive health	Develop a validated instrument to measure women's reproductive autonomy, that would apply to women in any type of sexual relationship and to women living in a variety of gender-equity contexts worldwide; that would capture the influence of other individuals in addition to the sexual partner and that could be easily inserted into standardized questionnaires, intervention evaluations, and clinical assessments.	03 subscales (1) Freedom from coercion (2) Communication (3) Decision-making	For each of the three subscales, a score is tallied, with higher scores indicating higher levels of reproductive autonomy.	Self-administrative survey conducted among women at 13 family planning and 6 abortion facilities in urban and suburban areas in the United States
Measuring women's empowerment: Indicators and measurement techniques (Biswas and Kabir, 2004)	Overall empowerment	Develop comprehensive indicators of women's empowerment in Bangladesh, methods for assigning weights for different indicators and sub-indicators, and a composite index for measuring the level of women's empowerment	11 indicators (1) Mobility (2) Decision-making power (3) Autonomy (4) Ownership of household assets (5) Freedom from domination (6) Awareness (7) Participation in public protests and political campaigns (8) Contribution to family income (9) Reproductive rights (10) Exposure to information (11) Participation in development programmes	The indicators were based on consultation of journals, books, and reports and an empowerment index was developed for each of them, based on the responses for each sub-indicator. A composite empowerment index consists of these different indicators. The weighting strategies include chi-square value method and opinion survey method.	Methodology only
Women Empowerment as Multidimensional Capability Enhancement (Bhattacharya and Banerjee, 2013)	Overall empowerment	Offer a quantitative measure for empowerment, viewed as a process of capability enhancement, and constituted of scores on three dimensions.	03 capabilities (1) Health (2) Knowledge (3) Autonomy	The regression coefficients from the MIMIC model work as weights in constructing the estimated capability scores as weighted averages of indicators. The empowerment index is the weighted average of the scores of the three dimensions	Primary survey carried out in two districts of West Bengal, India, spread over six different blocks of varied economic status.
Abbreviated Women's Empowerment in Agriculture Index (A-WEAI) (Malapit et al., 2017)	Agriculture	Develop a streamlined survey instrument that improves on the problematic modules and reduces interview time by 30%	05 domains (1) Production (2) Resources (3) Income (4) Leadership (5) Time	Multidimensional index reported at the country or regional level, based on individual-level data, represents the percentage of women who are empowered (i.e., achieve adequate4 achievements in 80% of the weighted indicators or more) based on the Alkire–Foster methodology. WEAI = five domains of empowerment (90%) + gender parity index (GPI, reflects intra-household inequality, 10%).	Survey data from the self-identified primary male and female adult decision-makers, aged 18 and older, in the same household from a pilot study in Bangladesh and Uganda
Measuring Gender and Women's Empowerment Using Confirmatory Factor Analysis (Williams, 2005)	Overall empowerment	Develop a new method for constructing measures of gender and women's empowerment with cross-sectional survey data.	06 dimensions (1) Makes decisions (2) Not modest (3) Makes small purchases (4) Visits women (5) Makes large purchases (6) Uses public transportation	Factor analysis estimates a weight for each indicator in each dimension of gender and the weights provide a way to generate a single measure for each dimension	1996 Matlab Health and Socioeconomic Survey
Women Empowerment Index (Varghese, 2011)	Overall empowerment	Measure women's empowerment by identifying their household decision-making ability, assessing their economic decision-making capability, and evaluating their freedom of mobility for giving recommendations to boost it.	03 aspects or dimensions (1) Economic (2) Household Empowerment (3) Social [physical freedom of movement]	Performance in each indicator is expressed a value between 0 and 1 in accordance with the construction method of the Human Development Index and the WEI is computed as a simple average of these three dimensions.	Household survey based on convenience sampling
Agency, Resources and Institutional Structures for Sanitation-related Empowerment (ARISE)–Study protocol (Sinharoy et al., 2022)	Water, Sanitation and Hygiene	Develop and validate quantitative survey instruments to measure women's empowerment in relation to sanitation in urban areas of low-income and middle-income countries.	03 domains and 15 subdomains (1) Agency - Decision-making - Leadership - Collective action - Freedom of movement (2) Resources - Bodily integrity - Health - Safety and security - Privacy - Critical consciousness - Financial and productive assets - Time - Social capital - Knowledge and skills (3) Institutional structures - Norms - Relations	Focused on empowerment at the individual, household, and community levels. Factor analysis and item-response theory approaches will be employed on data from each site to evaluate new items, test the factor structures, and to assess model fit and measurement invariance. Scale scoring will be performed by calculating sums and means for each scale.	Data collection is being carried out across five urban locations in South Asia and Africa to ensure that the survey instruments are valid and comparable across contexts.
Integrated model for women empowerment in rural India (Sharma and Das, 2021)	Overall empowerment	Develop an integrated model for women empowerment in rural India	03 dimensions (1) Economic empowerment (2) Social and human empowerment (3) Legal empowerment	A total of three factors were extracted based on the factor loadings values using exploratory factor analysis.	Own questionnaire developed based on a literature review including 20 statements related to empowerment of rural women suing a 5-point Likert scale.
Abridged Women's Empowerment in Nutrition Index (Saha and Narayanan, 2022)	Nutrition	Create a leaner WENI with fewer indicators without compromising on its ability to reproduce the nutritional empowerment scores and empowerment status	04 dimensions: (A) Knowledge (B) Material and social resources (C) Agency and autonomy 03 domains (1) Food (2) Health (includes fertility as a subset of health, relevant only to women aged 15–49y) (3) Institutions	The LASSO technique was used to identify a subset of indicators that best predicted the original nutritional empowerment score of individuals, as generated using the 33-indicator WENI. Same methods were used when computing both indices.	Data from two states, Bihar and Odisha were used as training dataset and data from three other states, Tamil Nadu, Kerala, and West Bengal, as the validation set.
Multicultural Psychological Empowerment Scale for Saudi Women (Moubarak et al., 2021)	Psychological	Construct a multicultural psychological empowerment scale for Saudi women in multiple cultures	04 dimensions (1) Meaningfulness (2) Competence/self-efficacy (3) Choice/self-determination (4) Impact	Pooled confirmatory factor analysis was using to determine the reliability and validity of the scale and the construct validity of fitness indexes was assessed.	Questionnaire applied to a sample of Saudi women from various age categories, different social, educational, and employment status, and geographical regions.

The measures in this group were mainly based on populations from individual countries, and often from selected population groups such as subnational regions, villages, or health centers. This set of publications also includes studies proposing measures to be employed in program monitoring and evaluation. All surveys had a cross-sectional design and most of them were especially designed to collect information on empowerment whereas others used secondary data or information from a data collection process with broader objectives. Two studies proposed indicators and metrics without applying them to any specific data (Biswas and Kabir, 2004; Lombardini et al., 2017).

The original WEAI and some of the measures derived from this index present a gender parity component, in addition to the results related to the women's empowerment domains (Alkire et al., 2013; Malapit et al., 2019).

We identified one study that used survey data from different years to test index invariance over time (Cheong et al., 2017) but it was not included in our final selection because the measure definition and validation (Women's Agency Scale) was presented in a separate publication already included in this review (Yount et al., 2016).

It is noteworthy that despite not proposing a new questionnaire for collecting data on women's empowerment, Mohebbi et al. (2018) tested the psychometric properties of the Persian version of the Healthcare Empowerment Questionnaire among Iranian women of reproductive age to validate the instrument for future use. A similar approach was used by Alquwez et al. (2021), who tested the psychometric properties of the Health Empowerment Scale Arabic version in measuring the health empowerment of Saudi working women. These two publications were not included in this review since they did not propose a new empowerment measure.

The number of domains comprised in each of the empowerment measures ranged from two to 11 and are also presented in Table 4, as defined by the studies' authors. As observed for the DHS-based measures, the names employed for the domains varied greatly (e.g., domains, dimensions, levels, subscales, indicators). However, given the specific characteristics of the measures, including women's life area, the purpose of the measure, domain definition processes, indicator operationalization, and data collection procedures, it was not possible to identify similar contents from the selected papers only based on their domains.

## 4. Discussion

This review of 36 publications that used individual-level data from different sources and distinct analytical procedures revealed the challenges in defining and measuring women's empowerment. The studies were all published from 2004 onwards and almost all of them analyzed data from populations from LMICs, most from African countries, and included mainly samples of women of reproductive age. Although representing an advance in the field of gender equity and development monitoring, a great heterogeneity of definitions and domains under analysis was observed, what seems to constitute a challenge in defining a measure suitable for comparisons across populations and over time.

Conceptually, the authors of the selected publications agree on several fundamental aspects such as that empowering women is not only necessary as a goal in itself, but also instrumental for development and that the construct of women's empowerment is very complex, multidimensional by nature, and context-specific. Nevertheless, the operationalization of the indicators took different paths that led to different measures of empowerment that often are not comparable. The multidimensional characteristic is used to rationalize the creation of either dimension-specific indicators (social, health, nutrition, political, and psychological empowerment) or composite measures attempting to be all-inclusive (Desai et al., 2022). Yet, the definition of the measures fails to translate the concept, especially those related to the definition of empowerment as a process of change in status from disempowered to (more) empowered and the importance of context specificity. We observe this in the selected publications, as many authors of the selected publications start by defining empowerment as a process, still, few elaborate on the need to use longitudinal data and follow-up of cohorts to capture the transformation from disempowerment to empowerment (Yount et al., 2016; Lombardini et al., 2017; Galiè et al., 2019; Dickin et al., 2021). That would be the only approach that aligned with the idea of empowerment as a process.

The different approaches, objectives, and theories could not but lead to a variety of empowerment dimensions and domains constituting each of the measures proposed. In turn, these dimensions were based on a wide selection of variables that were grouped and named differently in each study.

Frameworks conceptualizing women's empowerment in certain fields tended to be made up of empowerment dimensions that lead to an outcome or an achievement. Examples of such outcomes and achievements that are the product of empowering women are self-confidence (Soharwardi and Ahmad, 2020); reproductive health (Santillán et al., 2004); health and wellbeing through better use of water and sanitation (Dickin et al., 2021); improved nutritional status (Narayanan et al., 2019); and livelihood outcomes (Sharaunga et al., 2019).

Another struggle identified in the publications was deciding between a more meaningful and highly context-specific measure and a more generalizable measure that can be compared across settings. The first will be useful for a given country or region and is likely to be able to better capture specific issues that are critical to empowerment in that context. The second approach can help multi-country analyses and be key in global exercises of monitoring and evaluation and may be an important tool for supporting actions in a wider set of contexts. Yet, the validity of the measure in accurately measuring empowerment in different contexts is threatened.

Data sources were found to be critical in the creation of empowerment measures as they can either allow multiple country comparisons and, at the same time, limit the scope of the measure, or make it specific for a given context or objective but limiting the possibilities of comparisons. Measures based on standard surveys, such as the DHS, are based on variables from empowerment-specific modules, are easier to replicate and usually nationally representative, but on the other hand, are likely to cover fewer dimensions and be more general.

Most of the 12 studies that used these datasets employed similar analytical approaches, relying upon exploratory and confirmatory analysis and similar sets of variables, most of them encompassing decision-making, attitude toward violence, and social aspects of women's lives. The domains were usually defined based on statistical criteria. This approach, although allowing the method to be replicated, will vary depending on the set of countries included in the sample. Although not covering the process at the individual level, i.e., following the same set of women over time, a few studies attempted to capture changes over time at the group level using two or more DHS from a given country, such as Pakistan and Tanzania (Abbas et al., 2021; Mganga et al., 2021).

The publications examined presented abundant criticism of their measures and the measures of others. Firstly, regarding the DHS-based measures, the limitations include the fact that many of the relevant questions are answered only by women that are currently in a union, excluding single, widowed, divorced, or separated women and it is also limited to those of reproductive age (15 to 49 years) so that young adolescents, older women, and some of the most disempowered groups are excluded (Ewerling et al., 2017, 2020; Asaolu et al., 2018; Miedema et al., 2018). Besides the fact that the percentage of married women varies widely from country to country, this approach also assumes that women engage only in heterosexual or cohabiting relationships (Ewerling et al., 2017, 2020; Miedema et al., 2018). The studies that attempted to include unpartnered women by using sets of questions different from that used for the partnered ones, could not incorporate key questions such as those in the decision-making module (Kenya National Bureau of Statistics, 2020; Rettig et al., 2020). Also, the survey questions do not cover other sources of restriction to women's agency, such as parents and siblings, grandparents, family in-laws, kin, relatives, and others in the community (Kenya National Bureau of Statistics, 2020). Secondly, DHS covers just a few aspects of empowerment (Ewerling et al., 2017, 2020; Asaolu et al., 2018; Miedema et al., 2018; Kenya National Bureau of Statistics, 2020) not including, for instance, economic empowerment, sexual and reproductive empowerment, power relations outside marriage, participation in the community and public life, political engagement or influence, social and occupational leadership and positioning, freedom of movement and safety at the individual level, psychological empowerment, legal knowledge and rights, and participation, right to inheritance and property ownership is missing and might have led to sub-optimal measurements of empowerment, according to some of the included authors (Ewerling et al., 2017, 2020; Kenya National Bureau of Statistics, 2020; Rettig et al., 2020). Also, questions related to decision-making power are typically restricted to the domestic sphere and do not encompass decisions in the productive and economic sectors (Alkire et al., 2013). The attempt to overcome this limitation has been seen in surveys specifically designed for measuring empowerment in particular areas of women's lives. Finally, concerning the coverage of countries, it was mentioned that a limited number of countries are usually included in the analyses due to data availability, which makes the studies under-representative (Ewerling et al., 2017, 2020; Asaolu et al., 2018) and there may have a wide interval in the timing of the surveys (Miedema et al., 2018).

Those measures based on custom-designed surveys may be excellent for a given aspect as they may allow the theory to be reflected in the measure more adequately, but often cover only specific dimensions or are not easy to replicate or compare.

Our review fills a gap in the literature by summarizing studies intended to propose a women's empowerment measure, contributing to the understanding of different approaches and data sources used for this goal. Given that the evidence gathered spans heterogeneous literature regarding a concept that is very broad in scope, the use of a scoping review methodology is considered more appropriate. Also, we expanded the number of studies identified in previous literature reviews, were able to describe with more detail the methodologies underlying the measure operationalization, which had not been previously addressed, and reported the results separately by survey type given the specificities of the sets of studies identified (Desai et al., 2022; Nahar and Mengo, 2022).

Nevertheless, some caveats in the process need to be mentioned. Firstly, we did not include publications that proposed a particular empowerment measure but did not state that as the main objective of the study, otherwise addressing this among other goals, such as association with health or social outcomes. As a result of the eligibility criteria, we excluded those publications that used women's empowerment as an outcome, leaving out the ones addressing the impact of programs and interventions and therefore, possibly changes over time. It is noteworthy that no indicator included in the review focused on economic empowerment only and we hypothesize that this specific area might have been also captured by intervention studies, rather than using survey data. The measures identified during the review process collected data at the aggregated level rather than at the individual level (UNCDF, 2021; United States Agency for International Development, 2021). Another limitation is that we did not assess study quality, and some of the literature reviewed may suffer from methodologic flaws. Finally, we did not compare the results emerging from the studies. But these last two limitations are justified, at least in part, because our main objective was to explore the definitions and approaches rather than the results themselves.

## 5. Conclusions

Despite the difficulties and limitations, the fact that the literature has been accumulating proposed empowerment measures is very positive. To overcome the identified problems in the publications, the most important steps are to arrive at some consensus on a main set of constitutive dimensions of empowerment; agree on what would be the underlying information needed to estimate each of the dimensions; and ideally, propose a basic set of questions to be used in studies to collect the information defined above. The next steps would be to include this basic set of questions in standard survey families such as DHS and MICS; agree on a general approach to be used when deriving empowerment indicators that could be used in different applications; and agree on a single more general measure of empowerment to be used in multi-country analysis based on standard surveys. Also, regardless of the data sources, it is important to consider validation of the measures across contexts and consideration of within-country differences and measurement invariance (Asaolu et al., 2018; Desai et al., 2022). In summary, there is no single correct approach to measuring women's empowerment, and one needs to consider the broader purpose of this attempt, which might lead to different possibilities. The challenge is to find a balance between the need for a measure suitable for comparisons across countries or populations and the incorporation of country-specific elements.

## Data availability statement

The original contributions presented in the study are included in the article/supplementary material, further inquiries can be directed to the corresponding author.

## Author contributions

JC and AB conceptualized the study and contributed to the writing and reviewing of the manuscript. JC, GS, FH, and MM worked on the selection of studies and data extraction. All authors have reviewed and approved the final version of the manuscript.
